# Relationship-driven, family-centered care *via* TelePT: Reflections in the wake of COVID-19

**DOI:** 10.3389/fpsyg.2022.1030741

**Published:** 2022-11-30

**Authors:** Elena America Choong, Manon Maitland Schladen, Yvonne Beth Alles

**Affiliations:** ^1^School of Business, Technology, and Health Care Administration, Capella University, Minneapolis, MN, United States; ^2^Department of Biomedical Engineering, The Catholic University of America, Washington, DC, United States

**Keywords:** telehealth, physical therapy, pediatrics, TelePT, COVID-19, family-centered therapy, relationship-driven

## Abstract

In response to the throttling of children’s therapy programs precipitated by COVID-19 shutdowns, interest in the use of telehealth has increased among service providers at both the clinical and administrative levels. TelePT promises to be particularly appropriate in devising programs of on-going, therapeutic exercise interventions for children with neuromotor disorders. From the lay perspective, physical/physiotherapy (PT) which is seemingly characterized by the “hands-on,” and corrective approach to managing impairments, makes it a counter-intuitive candidate for delivery over telehealth. Over the past decades, however, PT as a discipline has increasingly adhered to a relationship-driven, family-centered model of intervention. This model is “hands-off,” figuratively if not always literally, and hence is not necessarily disconsonant with delivery mediated by telehealth technology. The current study explores in-depth the experiences and reflections of seven practicing therapists, on the impact of telehealth, telePT on the operationalization of relationship-based, family-centered methods into therapy. Interpretative phenomenological analysis was selected as the analytic method for understanding participants’ experience providing services using both distance and standard face-to-face practice modalities. Results identified eight principal themes emerging from participants’ descriptions of their experience of delivering therapy over telePT. Four of these themes correspond to the tenets of relationship-driven, family-centered care identified across four frameworks applied to pediatric rehabilitation. The remaining four themes focus on the particularities of the telePT modality and its viability in clinical practice. The ability telePT afforded to “see into the child’s environment” emerged arguably as the greatest value of the modality in patient care. It revealed to therapists so much that they did not know about their patients’ progress and, more strikingly, had not realized they did not know. TelePT provides a unique window into the child’s functioning in the hours he is not in therapy. Given its potential in parent–therapist relationship building, assuring the ecological validity of therapy programs, and the empowerment of families who seek it, telePT is likely to be part of the future of PT and one driver of its evolution as a profession. There is a compelling case to retain telePT modalities offering them alongside in-person formats for convenience, safety, and service quality enhancement.

## Introduction

COVID-19 brought about an unexpected, large-scale experiment with service delivery over telehealth. Though telehealth had been a technologically viable adjunct to standard clinical care for several decades (Libin et al., 2016), it was the need to enforce social distancing on a global scale that brought the telehealth modality into the spotlight (Doraiswamy et al., 2020). In the specific case of pediatric physical therapy (PT), there has been a system-wide and international focus turned toward telehealth, with a commensurate investment of resources to understand what works, what does not work, and what can be improved in the delivery of therapeutic services from a distance (Rabatin et al., 2020; Chivate et al., 2022; Daube Fishman and Elkins, 2022; Dostie et al., 2022).

Few physical therapists leveraged telehealth to provide clinical services to children prior to the pandemic (Hall et al., 2021) though the literature provides numerous examples of pediatric telehealth interventions shown to be, affordable, effective, and, in some cases, equivalent to in-person care (Olson et al., 2018; Shigekawa et al., 2018). According to a systematic review of pediatric telerehabilitation interventions published between 2007 and 2018, characteristics of effective distance services engaged parents in their children’s therapy, used a coaching approach, involved regular interaction over time, and centered around a defined program of exercise carried out in the home (Camden et al., 2020).

A home exercise program (HEP) to facilitate the practice of skills outside the clinic is a long-standing best practice of PT care. PT for children with chronic conditions, as typified by cerebral palsy (CP) is generally implemented episodically (i.e., in blocks of therapy of variable duration depending on the needs of the individual child), in an outpatient setting, and with additional practice prescribed for the child at least 1 h per week at home (Dodd et al., 2002; O’Neil et al., 2006; Novak, 2014). The timing of therapy is a key driver of positive outcomes (Gannotti, 2017). Consequently, carrying out prescribed exercises at home between PT clinic visits can increase the likelihood of the child’s getting the practice necessary to meet therapeutic objectives (Schladen et al., 2022). The home practice also uniquely provides ecological validity, the opportunity to train skills in the world outside the clinic, and to promote their transfer to real-life situations, as well as skill generalization (Steenbergen et al., 2010). However, adherence has been a major barrier to successful HEP (Taylor et al., 2004; Lillo-Navarro et al., 2015; Lord et al., 2018). Lack of confidence in how and to what degree a child adheres to his/her exercise regimen at home adds uncertainty to the measurement of therapeutic effectiveness and makes it difficult for the therapist to evaluate how well the prescribed exercise program is working for the child and to make adjustments if appropriate.

At first consideration, it is counter-intuitive that PT, a discipline characterized by a focus on therapeutic touch for the hands-on assessment and correction of movement (Sørvoll et al., 2022) should be compatible with delivery over telehealth. Over the past decades, however, PT as a discipline has increasingly sought to adopt a relationship-driven, family-centered model of intervention (Akhbari Ziegler and Hadders-Algra, 2020). This model is transdisciplinary (Baldwin et al., 2013) and focuses on empowering the patient and family to choose and engage in therapy that is consonant with their functional goals and can be integrated with relative ease into the family environment and routines. The family-centered model positions the therapist on an equal footing with the family and recommends the role of facilitator or coach, making information and resources available rather than prescribing regimens of therapy. This model is “hands-off,” figuratively if not always literally, and hence is not necessarily disconsonant with delivery mediated by telehealth technology. Therefore, PT services over telehealth (telePT) may be particularly appropriate for meeting the needs of children with motor disabilities that require regular follow-up evaluations and timely intervention if they have fallen off course.

### Objective

The purpose of the current study is to further expose and explore the salutatory effect that physical distance may have on building a therapist’s skill in the role of family coach to maximize the fit and impact of therapy in the environment where the child lives, learns, and plays (Hsu et al., 2021). The study has its origin in the reflections of seven pediatric physical therapists on the utility of telePT in more fully realizing relational and family-centered principles in the provision of care. These seven therapists were participants in the first author’s doctoral study (Choong, 2022) focused on the experiences of a broader range of health professionals (administrators, information technologists, as well as clinicians) relative to the unprecedented practice they had received in delivering services over telehealth, each from his/her individual professional perspective, during the 2 years of clinical business-as-usual disruption hazarded by COVID-19 shutdowns. This present work is a secondary analysis aimed at better understanding how pediatric physical therapists perceive the impact of telePT on their ability to deliver relationship-driven, family-centered care.

## Materials and methods

### Authors’ background and perspectives

EAC and MMS are human factors researchers, most recently in the context of game-based, home robotic home ankle therapy for children with CP. MMS is the parent of a child with a developmental disability and the godparent of a child with CP. Both authors have technical and management science backgrounds: EAC in biomedical engineering and healthcare administration; MMS in computer science, biomedical engineering management, systems engineering, and computing technology in education. EAC is a lead biomedical device reviewer within the US federal regulatory system. MMS has over 20 years’ experience in rehabilitation research with a focus on H.323 (internet-based) teletherapy, patient-centered care, and technology design. MMS served as EAC’s doctoral preceptor for her human factors work on remote therapy for children with CP. YBA is a professor of and expert in healthcare administration. She served as EAC’s doctoral advisor.

### Research question

Given the intensified exploration of telehealth service delivery methods over the past several years in response to the limitations imposed by COVID-19: How do pediatric PTs perceive the impact of telePT on their ability to deliver relationship-driven, family-centered care?

### Participants and recruitment

Semi-structured interviews were conducted with seven physical therapists experienced in the provision of clinical services to children and their families over telehealth. Of these individuals, five were frontline pediatric therapists and two were therapists principally engaged in program administration involving teleservices at the time of their interviews. See Table 1 for a profile of each participant.

**TABLE 1:** Participant profiles.

Frontline PTs, delivering services by TelePT	
***Hospital-based***	
Belinda	Belinda has provided in-person therapy for 8 years and teletherapy for 2 years, since the hospital’s ramp-up of telehealth services in response to COVID-19. She has held multiple offices in the Academy of Pediatric Physical Therapy and has clinical interests in cerebral palsy, as well as in vestibular rehabilitation, pelvic floor rehabilitation, cystic fibrosis, and traumatic brain injury.
Colleen	Colleen has been a therapist since 2005, providing therapy to children on both the in-patient and out-patient services and mentorship to junior PTs. Prior to the SARS-CoV-2 pandemic, she participated in several telehealth sessions to the United Arab Emirates that involved equipment recommendations rather than providing actual therapy to a child. Colleen began providing telehealth services to children and their families in April of 2020, at the beginning of the COVID-19
Kory	Kory came to teletherapy in 2020 with 6 years of experience as a pediatric physical therapist. She provides both in-patient and out-patient services and also see patients in multidisciplinary clinics, predominately burn. In addition to clinician-to-home teletherapy, she has experience providing remote acute care physical therapy for children admitted with COVID-19.
Sofia	Sofia has been a physical therapist for 17 years. Her induction into the telehealth therapist force came on the heels of COVID-19. Sofia’s clinical work focuses on infants and involves a great deal of hands-on therapy, working on gross motor skills for children who have had perinatal injuries that may eventuate in a diagnosis of cerebral palsy. The last years of her career have been focused on Early Intervention providing home therapy for children with developmental delays.
***School-based***	
Blake	Blake has been a physical therapist for 35 years with five of those years focused on services to children. He has administrative experience as the owner of an outpatient clinic and has taught at the university level. As the COVID-19 shut-down came into effect, Blake was engaged in providing therapy services within the school system. His experience with telehealth center around providing continuity of physical therapy to that population.

**PTs serving in education and administration of TelePT services**	

Kathleen	Kathleen serves as a professor and program director of a doctor of physical therapy program at a university in the Midwest United States. She administers a student-led *pro bono* clinic that had to pivot to providing therapy online after the COVID-19 shutdown. Kathleen had prior experience administering a remote service-learning program to Central America and has been involved in consulting in the context of global health physical therapy.
Megan	Megan shares experience from the perspective of therapeutic service delivery in Australia. Her primary role has been as a pediatric physiotherapist, but more recently has taken on the role of administrator. Megan practices in a very rural area and had made extensive use of asynchronous video-based materials in a coaching model of therapy for remote families prior to the rapid upscaling of telehealth in response to COVID-19. She has also leveraged internet resources to deliver training to other clinicians in the form of asynchronous webinars.

The five frontline therapists each had 2–3 years of experience with telehealth. All were working exclusively with children and were certified in pediatric rehabilitation by the American Board of Physical Therapy Specialties. Three therapists were employed in hospital-based clinical service delivery, both inpatient and outpatient. A fourth therapist also had experience working out of the hospital’s early intervention (EI) program where therapy services are typically delivered in-person in a child’s home vs. in the clinic. The fifth therapist was engaged in providing services in conjunction with his local school system.

The two participants who were serving primarily as therapy program administrators had between 2 and 16 years of experience with telehealth. Both had been practicing therapists before assuming managerial duties and brought additional experience in teleservice provision in academic, *pro bono*, and international contexts.

The first participants were identified from an initial convenience sample of therapists known to EAC and MMS through the pediatric hospital where they conduct research and through teleservice contacts of YBA. Subsequently, “snowball” methods were used to locate further informants based on current participants’ referrals. Prospective participants were made aware that their participation was entirely voluntary, as personal referral can lead to the individual being solicited feeling obligated to participate.

### Supervision and approval

The study was approved by and conducted under the supervision of the institutional review board of Capella University, Minneapolis, MN, USA (project number 2021-1578) and IRBear, the institutional review board of Children’s National Hospital, Washington, DC, USA (Pro000013680). All participants provided informed consent in writing.

### Interview guide

Sixteen primary questions were developed (see Table 2). Each question addressed a single topic regarding the therapist’s experience of using remote service provision techniques in their clinical practice in comparison to delivering services face-to-face. Questions probed perceptions of interaction quality of using telehealth, costs, how therapists engaged patients to do home exercises, and factors they identified as influential for the adoption of and persistence in technology-mediated home therapy. When the questions were addressed to therapists fulfilling an administrative role in telePT, respondents were prompted to share perspectives grounded in organizational considerations of quality, safety, cost, and benefit (the latter drawn from patient feedback) as well.

**TABLE 2:** Interview guide questions.

Please briefly share your experience doing therapy in-person in the clinic vs. virtually in the home.
How did you feel about the level of complexity in the therapy setup used in the clinic vs. that at home?
How did you perceive the effectiveness of the exercise routine your patient did during therapy in the clinic vs. at home?
How easy or difficult was it to learn how to conduct a visit using telePT?
What barriers and successes have you encountered in incorporating telePT into your practice?
In your experience, which type of telePT, synchronous or asynchronous, have you found more beneficial in your practice? Please explain.
What types of training have you had in telePT?
What feedback, if any, have you had from your patients using telePT? Do you feel like they felt comfortable using it? Did they feel it was safe to be using the system?
Please briefly share your experience regarding the interaction quality of telePT.
Did you feel that you were able to communicate the instructions for the home exercise program to your patients over telePT as well as you did when you were with them face-to-face?
Please briefly share how telePT has (or has not) provided cost and time savings for you.
Has telePT improved or diminished the quality of care you provide for your patients?
Do you think telePT has increased or decreased your ability to monitor your patients’ adherence to their home exercise programs?
Thinking ahead 6–8 months, do you anticipate you will continue to provide patient care *via* telePT? Why or why not?
Is there any other information you would like to share about your clinical experience using remote delivery of services in comparison to face-to-face therapy sessions?
What has been your greatest win with telePT services?

### Interview conduct and verification

Hour-long, semi-structured interviews were conducted *via Zoom Meeting*,^1^ a well-disseminated teleconferencing app the use of which had become routinized for each participant in both clinical and administrative interactions in the aftermath of the COVID-19 pandemic. Interviews followed the pre-developed guide but allowed participants to extend or refocus the discussion as it related to their personal experience with distance service provision. The first author (EAC) led each interview; the second author (MMS) was present for a subset of interviews and asked questions for clarification. All interactions were video recorded using the native Zoom functionality. Audio files were transcribed verbatim by a professional service and transcripts were verified by the authors. Subsequently, each participant was provided with the verified transcript of his/her interview and asked to provide revisions or clarifications as they felt better represented their experience with remote service delivery.

### Analyses

Interpretative phenomenological analysis, IPA (Smith et al., 2009), was selected as the analytic method for understanding participants’ experience providing services using both distance and standard face-to-face practice modalities. IPA provides a framework for exploring each participant’s experience individually and subsequently creating cross-linkages across participants, exposing similarities and differences to create a model of the phenomenon of interest. Participant-elaborated transcripts were uploaded to NVivo 12, qualitative data analysis software for coding. The initial code book was developed from the key concepts of the interview guide. These codes were iteratively elaborated as open codes (Straus et al., 2009) based on experiences and perspectives shared by participants that researchers had not previously encountered in the literature and incorporated into the original interview guide.

Coding was begun from the first interview. Coding interviews as they were completed facilitated the hermeneutic process essential to IPA, crystalizing concepts for analysts that they were then able to use to expand (or prune) the interview guide to better focus the questioning with subsequent interviewees. EAC performed the initial coding on each interview. MMS reviewed the interviews and initial coding probing for additional themes in participants’ shared perceptions and experiences. It was from these additional themes that participants’ experience of the impact of the telehealth modality on the provision of relationship-driven, family-centered care emerged. Authors resolved disagreement about the interpretation of themes through discussion and consensus.

## Results

RQ: How do pediatric physical therapists perceive the impact of telePT on their ability to deliver relationship-driven, family-centered care?

### Themes

Eight principal themes emerged from participants’ descriptions of their experience of delivering therapy over telePT. Four of these themes correspond to the tenets of relationship-driven, family-centered care identified across four frameworks applied to pediatric rehabilitation (Akhbari Ziegler et al., 2019). The remaining four themes focus on the particularities of the telePT modality and its viability in clinical practice. In the sections that follow, each theme is introduced with the explanation and representative data (quotes) presented. Therapists’ responses to the interviewer’s prompts provide quotes illustrating each theme. Since the discourse was conversational, stopping, starting, branching, and returning to earlier thoughts, the authors have used punctuation to replicate the natural flow. Where there is ambiguity, clarifying words not part of the original quotation are inserted in brackets.

#### Sensory reorientation for physiotherapy services over telehealth

TelePT required a sensory reorientation: from the kinetic and tactile (doing and touching) to the observed and articulated (seeing and speaking). It involves moving from the implicit spontaneous communication in-person in the clinic to the explicit and planned visual assessment of the environment into which the therapist has entered *via* tele-technology and communication with the family on the other end to operationalize the therapy interaction for the child.

Belinda described the phenomenon in this way.

*I’ve done in-person therapy for eight plus years and virtual therapy for two years now. I said they’re two very different experiential pieces because you rely a lot more on very different sensory cues because in the clinic, I can use a lot of tactile feedback and I can really get a feel for what I’m [feeling] and also hearing instead of just visualizing. Whereas, I rely a lot on visualization when I am doing telehealth therapy. Also, [I need to rely] a lot on questions of both my patients and their family, which is a lot more challenging!*

The shift from in-person delivery of PT services where therapists could leverage the full range of their senses in providing care to delivery over telePT where the visual and verbal senses became the principal channels for clinical decision making introduced a fundamental shift in practice.

#### Physiotherapy services over telehealth in building the therapist–family relationship

The first and essential step in relationship building, according to relationship-driven therapy models, is interviewing the family, learning to ask the right questions, to understand what their goals and priorities for therapy are, since these are the goals that will likely be able to be accomplished.

INCREASED IMPORTANCE OF ASKING QUESTIONS (INTERVIEW SKILLS)

Belinda shared the challenge of gathering all the information she needs as a therapist through verbal interaction with the family.

*I think to have all of your information come from an interviewed style versus seeing it on your own can be difficult for some of our standardized measurements and tests that we would want to get done.*

Belinda recognizes that a workable approach to clinical assessment at a distance is not the same as the one she uses in the clinic.

DISCOVERING RESOURCES FOR THERAPY IN THE HOME

One practical goal of questioning the family is to discover what resources are available in the home to carry out a therapy session. Colleen contrasted the typical scenario for clinic-based PT vs. setting up a home session at a distance.

*In some ways, that [doing therapy in the clinic] makes it simpler because everything is at your fingertips. It’s right there, and you know your own equipment. I can plan the day before what I’m going to use. [On the other hand,] sometimes another therapist might be using that piece of equipment. I have to time around who’s going to be on the treadmill at what time.*

*Then with telehealth, you don’t know necessarily what they have at their house.*

*In fact, I was seeing a kiddo for weeks before I knew they had a treadmill! Another kiddo had a full gym in their basement with floor mats and all kinds of equipment. I think that with telehealth, you have to ask a lot of questions about what the home setup is like, which can feel strange because you want to give people their own privacy and not make assumptions about what they have.*

*I think it can be really helpful, now that I’ve done it, to ask more open-ended questions like, are there any other rooms in your house that would be helpful for me to see? Or do you have any equipment in your home that’s for exercise?*

Colleen learned that open-ended questions are an effective technique for learning the home landscape.

DISCOVERING THE FAMILY’S EVERYDAY LIFE

Building a therapeutic alliance involves understanding how the family’s life unfolds in their environment as well as what that environment contains that might be analogous to what the physical therapist would use for therapy in the clinic.

Kathleen shared her experience building therapeutic alliances over telehealth.

*It’s very easy to build that therapeutic alliance [even over telehealth] and get to know the people, but I need to ask questions to know what their everyday life is like. Also, can they describe a typical day?*… *I always talk about your child and ask them, what’s the best thing about their child. “Tell me about the really cool things. Tell me about some of your challenges.” That’s how we start to collaborate through this medium of telehealth to start to develop a plan.*

*Do they believe in practice or not? Part of my educational message then if they don’t believe in practice, I’m not going to say, “You need to practice this three times a day.” I might say something like, “Well, okay, practices and everything, but here are some strategies you can use to position your child as they’re interacting with their other siblings while you’re doing the wash or doing the dishes or cleaning house, this, that, or the other thing, or while you’re away at work and someone else is taking care of your child.”*

Kathleen finds out what the family’s goals and preferences are and casts whatever knowledge she might have to share in their frame.

POTENTIAL FOR BUILDING A STRONGER FAMILY-PT CONNECTION *via* TELEPT

Colleen shared her experience of the possibility of building a stronger connection with the family over telePT than she might typically expect in the clinic.

*I think in some ways, it [telePT] promotes more interaction because you’re looking at each other face-to-face. I don’t know if you know the bubbles of space determination, but it’s like, if someone is face-to- face with you, that’s intimate. If they’re at arm’s reach, that’s personal, and then if they’re past that, that’s public. There’s something about having your laptop or your phone in your space that feels a little more intimate. Because we’re looking at each other right now, I’m not going to just do this or that. It would feel really rude.*

*I think for some of my families, I actually got more interaction with them because that is the expectation of communicating with someone this way versus if you go to a doctor’s office, they may just show up and tell their kid to join me, but they may get on their phone and start doing whatever and not really interact and engage all that much. Because they had to facilitate what their child was doing [over telePT], then I felt like some parents interacted a lot more. At the same time, I do feel like some of the families, it was awkward.*

*Most of the time in the clinic, I think that we have a good rapport with patients. In clinic, I’m there with as many as two or three other therapists. There are sometimes other families there. There are sometimes kids with behaviors that are loud and persistent and may be distracting. With this [telePT], you and I are having a private conversation, you don’t have to worry about what some other family is going to overhear you saying, and you may feel a little more comfortable sharing embarrassing things, I think, especially because you’re also in your home.*

*I feel like some parents were able to say like, “Oh, we’re really struggling right now. I’m really struggling with sleep and eating and all these things. Nothing is going really well.” We had a lot of therapeutic conversations and not solely physical therapy. I don’t know if parents would have shared as much of that in the clinic.*

For some parents, though not all, engaging with the therapist over video media may be a facilitator of relationship building.

USEFULNESS OF PRIOR IN-PERSON CONNECTION

Several therapists perceived that having an initial, in-person introduction to the therapist was useful in establishing a subsequent, ongoing relationship over telePT.

Megan shared her experience.

*I’ve thought about this a lot, because if I’ve had a connection, a face-to-face connection with a family, we’ve started to work together and we’ve made a connection, both of us are very comfortable following up with Zoom, because I follow up with phone calls anyway all the time with families and we’ll sit and discuss things. Zoom is just another nice next level to go where I can actually also see how the child’s doing, which is fantastic. And that works well.*

*However, if you have not made a connection with the family, I’m having more difficulty because the family is not comfortable. They don’t know me and they’re not as. They say they’d like to, but I have one family right now that was referred to me and they live in Canada and they really love me to help them, but it’s really hard to get to first base, to get that first meeting up and going. And I think it’s because of the discomfort and they don’t know how it’s going to go and how it’s going to look and how it’s going to feel. Whereas people who’ve already met me and have that connection, it’s worked really well. That’s the only downside I have found in face-to-face services, is that it’s pretty hard if they don’t really know what you’re doing or going to be doing.*

Sofia contrasted the experience of connecting with adults and connecting with children over media.

*I think with kids who I already know, I had a relationship before I moved to telehealth, that was a little bit easier. Meeting families through the computer for the first time was fine with the adults but sometimes hard to get the little ones to engage with you.*

*I feel like the kids who already were Face Timing with grandparents that don’t live local, were very used to this as an interface. They’re like, “Oh, yes.” Especially some of the kids who knew me before. “Miss Sofie’s on the computer. Hi.” That was completely normal for them and they were still able to play*

*Whereas some people[children], you clearly were like a TV show, and they didn’t understand that they could participate with you and that was confusing for them.*

A child’s prior experience with interactive video media primed them for a productive interaction over TelePT.

IMPORTANCE OF COACHING SKILLS IN RELATIONSHIP BUILDING

Megan shares her experience that enacting the coaching process is useful for establishing the therapist–family relationship that is essential to successful telePT interaction.

*I have so much experience coaching, so I always start a family out by interviewing them. I do a lot of observational assessments, which is easy to do for me because of my experience on a video versus in person, because I’m observing. And parents, once they know that I can get everything I need by observing, especially after the initial assessment where I might be doing some manipulation, and then I demonstrate using a doll which I can do on telehealth, they’re comfortable. But as I said before, on the first visit, you really need to connect and they need to appreciate that you’re not going to need to do all hands on, and then their comfort level and their confidence in that you can see what’s happening over telehealth increases.*

Megan’s perspective underscores the essential dissonance of therapy with no hands-on involvement of the therapist and this is only resolved through relationship, which in turn fosters a family’s satisfaction with telePT.

DISCOVERING THE FAMILY’S GOALS

*Understanding a family’s goals for a child’s function is important to building the family-therapist relationship. Sofia shares her experience of family versus clinical perspectives.*

*Usually, the kids who have a goal for themselves, or a family has a goal for that patient, then they see the progress and they’re more willing to work on it. A lot of times, our ability to tie in, why are we working on weight shifting and tummy time? Why is that important for a two or three-month-old to be able to do and figure out that movement?*

*If we can explain to a family that that’s what walking is and that’s the weight shift and that we’re learning and practicing all these skills because here are the skills that help later in life, I feel like then they have buy-in and they want to practice that as well. Definitely tying it into the functional skills and not just saying, “Oh, there’s a little asymmetry, one side shorter than the other, we need to focus on that.”*

There is dissonance between clinical goals as therapists think of them and functional goals that have significance to the family. Sophia provided an example of how to transform a clinical goal into a functional goal that a family supports and is willing to act on.

#### Family empowerment

Family empowerment, giving the family the skills to work toward achieving their goals and make increasingly better-informed choices about what therapy should look like is a component of relationship-driven, family-centered therapy. Kathleen described the principle this way:

*Your job then is to try to teach the parents to work [you] out of a job because they’ve got to provide support at certain levels and then give them [the child] less support so the child can start to use their levers and their body more independently.*

Megan provided an example of family empowerment from her own telePT practice.

*We live in a rural area, so we have quite limited services. And I did use telehealth on several occasions with clients who called in, families, and I used the telehealth ability to just walk them through what they needed and where I thought they could best get their services met in a very rural area, and with a couple of ideas, and then I’ve done a lot on coaching. I use a coaching strategy to get them to come up with their own solution. And telehealth works very well with coaching.*

A coaching approach, where the client is guided in coming up with their own solutions, is synonymous with the idea of empowerment.

VARIABILITY IN FAMILY’S PERCEPTION OF BEING EMPOWERED

Participants experienced different levels of acceptance of empowerment in their families, however, from enthusiastic uptake to absolute rejection. Colleen provided an example of ideal family empowerment that she facilitated *via* telePT.

*Probably the kiddo that you saw with me. He has just done so well, and watching the whole family get involved. I taught her [the mom] how to use a stander which is like a special piece of equipment that a child can stand in. I found one for them at a nonprofit. They went and picked it up. We looked at it together over telehealth. I taught her how to adjust it. She started using it with him and he didn’t like it, and so we did some unorthodox stuff where I was like, “Okay, go ahead and put him in it, and have the boys around,” and so his two brothers. The brothers were both there and I was like, “Okay, roll him across the floor from Hudson to Jonathan.”*

*Like they played catch with him in the stander and he loved it. He was so excited. They were dancing with him, swirling around and stuff. Just to be able to give parents tools to help make something that feels new and different and scary to make it fun, and family-friendly and kid-friendly. He got to where he was using a stander like an hour or more a day. That really helped to improve his ability to take weight through his legs and build up the muscles in his legs, and also to just improve his joint range of motion, because he couldn’t get his knees all the way straight.*

*With the device, there’s like a pad that you can push against the leg and it pushes it more straight. What we did was gradually. She [the mom] would send me a video or a text picture of it and I would draw on it and be like, “Hey, move this this way and move this this way.” He initially was standing cockeyed and we got him more upright. She would make the changes and send it back and I would be like, “Yes, that’s exactly what you want!”*

Colleen’s family demonstrated a high degree of empowerment. Blake relates an experience at the opposite pole.

*A lot of the parents, when I talked to them and said, “Okay, you’re going to be my hands and I’m going to watch and you’re going to do,” they’re like, “No. We’re done here. We’ll just wait.” A lot of our parents of our children really had opted out and that’s part of the law. The law is they’re the guardians. They do not have to. We have to offer it. What we did was, I would make an appointment every week and say, “Okay, this is your appointment time. Does it work for you?” They’re like, “Yes.” I’m like, “Okay.” I have to offer it. You don’t have to accept it. For 18 months, two-thirds of my caseload I never saw because the parents did not wish to participate.*

*Well, what I would do is, I would call them every week and some may say, “Hey, we’ve got our time.” Then I would send them an email saying, “Hey, here’s our day.” Then most of them never showed up. They agreed to it because they didn’t want to tell me no. They agreed to a time but they just never answered the Zoom call.*

*I can’t make these parents do anything that they don’t want to do. I get it. My parents, when you work in a certain environment that’s socio-economically so impoverished, therapy is so low on their list of what is important at this moment in time. They’re worried about rent, they’re worried about other things. As much as I was frustrated, I really was like, I don’t know that I could walk a day in your shoes because you have so many other stressors that the least of them is me. You worry about whether or not your child is going to get their physical therapy session today. It’s so not on their radar.*

Blake’s experience demonstrates the challenges faced by marginalized communities even if they have the necessary technology to engage.

IMPACT OF AN EMPOWERED PARENT ON PROGRESS TOWARD THERAPY GOALS

Therapists generally perceived that empowering the parent resulted in accelerated progress toward therapy goals today. Sofia relates the experience she had working with a nanny (parent surrogate) over telePT in the case of a long-time patient.

*I had one little boy that I’ve been seeing for a number of years, and I struggled to get them to bring the gait trainer or bring the walker into therapy, they just wanted me to do exercises with him.*

*He’s really a dependent kid who’s not initiating any movement on his own. He made huge gains and he had a really focused nanny that was with him all the time and just wanted to learn and wanted to do these things. He ends up meeting all of the goals that we had for several years for him just by doing it. When he came back to the clinic, it was like, we’re not making any progress anymore in the clinic more than we were in telehealth.*

*Telehealth was a better fit for the family because the nanny was doing it with him every single day. I feel like just getting to see people’s homes and see, “Okay, what’s working for you? How can we incorporate and adapt these exercises to really work in your day?”*

#### The value of seeing into the child’s home environment: Ecological validity check

Therapists expressed astonishment at how much value they derived from the affordance of seeing into the patient’s home when connecting over telePT. Seeing into the home continuously revealed misconceptions they had inadvertently formed in five main areas as enumerated below.

1-WHAT EXERCISES A CHILD COULD/COULD NOT DO

Sofia related:

*I think my kids who had been coming for years and expecting that hands-on therapy, it was really challenging for them. It was also pretty eye-opening for me. Some of the stuff is like, “Oh, I thought you guys were independent with some of these exercises and you are not.” That was really eye-opening for me of how we need to step back and, again, maybe make some of the exercises a little bit easier so that families can actually do them.*

2-FUNCTIONAL SKILLS A CHILD DID/DID NOT HAVE

Sofia provides a further example of how she prefers to ascertain function in the child’s home environment.

*I have one kiddo who I never saw in telehealth, but I’m starting to see now for an outpatient. I recommended even if we stay with weekly therapy, if we have one or two additional telehealth visits just so I can see the setup at home, because she’s a 17-year-old and I think a lot of it’s posture with her. Sitting in different chairs at home, and I don’t think I’m going to get a good sense of that in the clinic. She sits up perfectly in the clinic chairs and equipment. Getting to see how kids function and look in their home environment is so important for us, really making a difference in our families.*

3-THE APPROPRIATENESS OF PRESCRIBED HOME EXERCISE GIVEN THE CHILD’S ENVIRONMENT

Belinda reflected on how the variety and richness of equipment available in the PT Gym can mislead therapy.

*When you’re in the clinic, you’re afforded a lot about opportunities to utilize everything. Look at where I am now, I’ve got 10 swings and three fair balls of different sizes. I’ve got a basketball hoop, and that’s just in a quarter of our gym right here. Sometimes that is extremely helpful. It gives you all the opportunities in the world but it can also be almost choice-paralyzing because you almost have too many things to work with and it’s almost too distracting.*…

*A lot of this it’s a false environment. It’s not something that they can take home. I do a lot of things here that aren’t applicable at home versus in virtual say, it was really interesting to me that with telehealth, or teletherapy, we were really able to see a lot of what patients actually had at home and what they would actually be able to use and do with the equipment and environment that they had.*

*I think maybe my adherence has gotten better since pandemic and since the initiation of virtual, because I think I’ve learned from virtual that my initial exercise programs are probably a bit too ambitious and I think I gave patients and families too much to handle. That also caused when you have too much to do, you’re just not going to do any of it. You give five exercises, you are not going to touch any of them, but if I give you two, maybe you’ll touch two exercises. I think I actually have improved a little bit from that realm of learning from my own self.*

4-Clues as to whether the child was actually doing the home exercise program

The interaction with a broad range of family members when therapy is *via* telePT increased information available, as Colleen relates relative to knowing whether a child has done his/her assigned home exercise.

*They [the patient or parents] don’t like to disappoint you. They don’t like to say we didn’t do it. I always tell my families, I’d rather you tell me you didn’t do it than lie to me because I’d rather know you didn’t do it and I’m not going to be mad or upset, I just want to know.*

*I feel like when there’s more family members at home and you catch them virtually, especially when they’re siblings, they’ll tattle on each other and they’re really good at it. I know a lot of siblings who will be like, “Joey, didn’t do his homework.” From that aspect, I feel like I get a little bit more honesty and candor because, and maybe it’s just they’re in their own environment and I don’t know, maybe I put off judgy vibes when I’m in person, but I think maybe it’s the fact that they’re not coming to see me and maybe they just feel like they can say more often like, I didn’t do it this week or maybe it’s the fact that they’re in their own environment.*

*I feel like maybe I just get a bit more truth or somebody tells on them and that happens, I will say that has happened to me a lot during virtual therapy that somebody will yell that out when I ask it.*

5-OPPORTUNITIES FOR EXERCISE AT HOME BASED ON THE DISCOVERY OF FACILITATORS THE THERAPIST HAD NOT KNOWN WERE THERE

Kory described the richness of information available in the home to guide therapy.

*I do think one benefit of a virtual visit is you can look at the patient’s home setup and give them their homework directed toward their environment versus if they’re coming into the clinic, you don’t always visually see what their home setup is. I can actually see their home setup and see how tailor my exercises to their home. Versus when they come in clinic, I’m providing them with exercises that work in our clinic setting but might not necessarily work well in their home. Often, when you’re telling families things to work on at home and patients things to work on at home, they’re focused on what you’re telling them.*

*They might not be thinking like, “We’re doing this from a bench in the therapy gym, and we don’t have a chair that’s that height at home.” They might not be thinking of that at the time versus when I see them on telehealth and can see what height the chair is or what height benches they have in their home. I can tell them, “Work on this in that area.” I think overall, it’s definitely increased their adherence.*

Therapists found being able to better assess what is going on with the child’s program when not in a therapy session was an extremely useful facilitator of increasing the precision and quality of care.

#### Equal partners: Family and therapist

Therapists shared experiences affirming the logic and utility of establishing a stance of equity between the therapist and the family particularly relevant to the telePT modality.

PARENT INTERACTION ESSENTIAL FOR WORKING WITH YOUNG CHILDREN OVER TELEPT

Young children at a distance can get out of control unless the parent maintains a presence. Colleen observed:

*I think for my friends that did adult therapy, it’s a little simpler to just me say to you, “Hey, can we do your exercises now?” You say, “Yes.” [laughs]Kids don’t always do that. I had a couple of times where they left me with a kid. A phone propped up somewhere, and the kid would move and it would fall down. I’d be like, “Hello? Anyone? Anyone up there who can pick me up?”*

Kory remembered:

*If there are siblings or dogs running through the screen and you had to be like, “Focus on me,” and really get the kids’ attention, occasionally that’d be hard. Especially if they left the room and you’re left with a blank screen, then it’d be obviously difficult to communicate and bring them back in.*

THERAPIST AND FAMILY WORK AS A TEAM

Sofia relates an experience of working with a mom during telePT.

*I think we just had to go with the flow. Sometimes you’re not going to be part of the session and that’s okay. Sometimes you’re just a voice in the background saying like, “Mom, why don’t we try to move the puzzle pieces up here?” You’re not playing with the kid and that’s not fun for you as your PT job. but still effective. Some kids just weren’t going to engage and do that play social relationship with you in the computer, but if the parent was the one doing the play, that was still okay, you were still meeting your role and why you were there.*

Sofia and the child’s mom partnered to deliver his therapy that day.

PARENTAL SKILL ACQUISITION

Therapists experienced real growth in parents’ ability to effectively implement therapy through the partnership they established over telePT. Colleen related a particularly satisfying experience.

*You got to see one of my patients over telehealth, and that mom has become– She’s so good at doing so many things. She’ll text me videos of him and say, “Oh, I took that concept that you showed me and I used this space, and these toys, and whatever.” I feel like opened up, how effective I can be as a therapist, because I think I really focused more on the child and the changing child.*

The mom demonstrated ongoing, innovative thinking about how to meet her son’s needs.

#### Bidirectional observation for real-time coaching over physiotherapy services over telehealth

Therapists appreciated the ability to demonstrate maneuvers to parents *via* telePT and then observe and correct the parent’s return of that training. Colleen reflected on the process.

*Being present, having the parents be able to see you, you see them, I think it’s super helpful for being able to provide feedback in real-time with how they’re facilitating things. I think the majority of my patients need some form of physical assistance.*

*Part of the learning curve for parents is how can I facilitate higher-level movement and not just do things for them? Because a lot of parents will lift and move their kids versus supporting them while the child stands and helping them to take steps and things like that. I think being able to be present in real time and say, “Hey, see how they’re doing this? Okay, that means that this is happening or this muscle is not active.” We want to try and promote, so I can really share like how to change what they’re doing to make it better.*

The parental learning curve presented a challenge to the therapist’s hands-off coaching skills.

CHALLENGE OF VERBALLY DESCRIBING MANEUVERS TO PERFORM

Colleen described the difficulty of verbal coaching necessitated by the distancing medium.

*A couple of times I would be like, “Okay, so not what you’re doing. I want you to stop and look at what I’m doing.” Whereas in the clinic, I feel like I could just go over to them and physically show them what to do. Sometimes I had to be pretty blunt and direct, especially when the parent was– sometimes the parent would be doing what they think they’re supposed to be doing.*

Belinda shared her frustration at not being able to intervene physically—ever—over telePT.

*If we’re only seeing a patient through a screen – because sometimes the screen slipped and what I thought was their right side is actually their left side! – and sometimes family is having a hard time putting their hands where I want them to put their hands. When they’re in the clinic, I can put their hand exactly where I want them.*

The potential for error inherent in the limited sensory input available over telePT was disconcerting to therapists.

CHALLENGE OF PARENT’S FOCUS ON THE WRONG DETAIL

Sofia related that parents also experienced frustration with the inability to abandon virtual coaching and redirect with physical coaching.

*In those families who really want, who are so focused on exactly where their hands should be, that that piece, showing them was a little bit more difficult. I have one little girl who mom is so focused on exactly where my thumb should be versus my finger. It’s getting her to see, well, it matters how this child’s body looks, not necessarily exactly where your hand is going to be and that she just would get so frustrated with it and I think push the little girl a little bit too much past the point of her comfort with working.*

The concern was not only the parent’s frustration in learning with verbal-only coaching but also the discomfort of her child.

Complexity of Demonstrating over TelePT

The complexity of demonstrating over telePT that therapists experienced was in the area of staging and clinical planning, not in operating the technology *per se*. Kory described her experience.

*Technology-wise, I actually feel like it was pretty easy. I felt like the technology was simple to use. We used Zoom right away. We already knew how to use a video camera just from having your cell phones and doing Skype sessions outside of work. From a therapy standpoint, I do think it was hard. One thing that was difficult was figuring out how to change from conversing with your patient to demonstrating exercise. You had to often have a setup of how you would demonstrate something in standing and then something in sitting and just know how to change your camera angle quickly, or have everything set up around you ahead of time so that your session went smoothly.*

Planning and rapid response to shift viewpoints were the principal issues of technology complexity in conducting a session over TelePT.

#### The dilemma of no hands-on (ever) over physiotherapy services over telehealth

Kathleen shared her perception of the inability to touch the patient over telePT.

*Again, [say] you’re focused on kids with CP. I think the hard part for the therapists is to not feel the tone or the spasticity, and then, working with the parents, to give them some strategies to break that down. I think it can work well. Again, oftentimes, the therapists have this fear of something [undetected due to their inability to touch the patient]. Certainly, the patient has to feel that, and their families feel that they’ve got a connection to you as the PT, and that this is a valuable service because they’re paying their hard money. That can be a challenge.*

Fears surrounding the lack of ability to touch the patient include both potential harm to the patient and harms to the trust relationship established with the family.

HANDS-OFF IN THEORY

Kathleen articulated a representative theoretical perspective.

*In a telehealth platform, we are not touching our patients, we’re laying our hands on the patients per se, which is physical therapy. It’s so physical that that part is a little bit different. How do you do that? For peds, oftentimes we’ll use little dolls to talk with parents about, first of all, get to know the– We try to use an I-CAN-DO model, interview the parents and the family, what is their situation like, collaborate with them on things, and then end with them saying, “Ah, yes, I can do this.”*

Therapists generally agreed with the practice rationale for hands-off coaching though not without reservation.

Hands-off in Experience

Kory’s thoughts reflect the general ill-ease among therapists relative to the inability to have hands-on with the patient over telePT.

*I still felt like I could provide gross motor treatment in the clinic setting and that home setting, but there’s benefits for in-person as well because I couldn’t do hands-on treatment [in the virtual environment] to demonstrate for parents. A lot of times, parents would be like, “Am I doing this right? I’m not sure if it feels appropriate.” When the kid’s in person, you’re able to stretch the kid or do something with the patient and then have the parent try it so that everyone knows they’re doing the right thing.*

Being sure they are doing the right this is the essence of the therapists’ concern over a modality that does not have the ability to afford hands-on interaction, the current state of affairs for telePT.

#### The continuing evolution of physiotherapy services over telehealth in clinical practice

Therapists stressed that the learning and advances in telePT praxis spurred on by the necessity of COVID-19 should not be lost. Of particular note was the evolving nature of PT and the logic of not limiting any tools at the clinician’s disposal, specifically those brought into being through telePT as well as those long practiced with patients in-person in the clinic and home (especially in the context of EI).

THE EVOLVING PT PROFESSION

Kathleen shared her perspective on PT as a profession in a time of rapid technological change.

*I think the pandemic really gave us the push off the curve to fully hit the road running and say, “This [telePT] is a real possibility for practice.” I think this is a new mode of delivering services*… *It’s really re-envisioned how we interact with our patients. We’re training students for our profession that is ever-evolving. Really, how do you train someone for a job that really isn’t clearly defined yet? We train them how to think, how to problem solve, how to be innovative and creative, and yet still maintain the basics of excellent patient care and therapeutic relationship building. Within our American Physical Therapy Association, the education section states that every PT and PTA or physical therapist assistant is an educator. As we look at our roles as physical therapist, we are administrators, we’re clinicians, we’re consultants, we’re researchers and we’re educators.*

PT is well-positioned to grow from the addition of telePT to its clinical armamentarium.

THE DESIRABILITY OF HYBRID PRACTICE

Colleen provides a thorough rationale for the leverage of in-person and telePT modalities as best suited to the needs of individual patients.

*I feel like now that we’re not in that acute phase of the pandemic and things are opening up a little bit more, it’s been nice to– some families prefer to do like two visits of telehealth and two visits of in-person. That’s been a nice mix because then we can do some hands-on experience together in the clinic. Then we can also focus more on that home exercise piece for the off weeks.*

*I don’t think it [telePT] would be really anyone’s consistent model of service delivery. I think most people will choose at least a mix of in person or telehealth, with the exception of a few really immunocompromised kids are not feeling comfortable enough to come into the clinic. I have a few kids with cancer who, when they’re really neutropenic, they’re not feeling comfortable coming in. We do telehealth those days and then when their cancer a little bit better, they come in for in person.*

*Then also too being at a major children’s medical center, we see a lot of kids that come from far away, and so being able to touch base over Zoom [is ideal]. Before I would’ve been like, “Oh, you guys are fine. Just call me and we’ll chat if there’s any issues but I think you’re good to stop therapy.” Whereas now, I might say, “You know what? I’d actually like to see you [over Zoom] once a month for the next [few months] and see how things are going.”*

*I think it’s made it easier to monitor. In fact, for some of my families, I’d almost have a hybrid where we meet once a month online instead of coming to the clinic so that I could do that purpose of just having them show me what they’ve been doing at home and reinforce that it’s possible to do all this stuff at home.*

*Yes, I can see myself continuing to use it [telePT] both to assess the effectiveness of what of my parent education, like how much are they understanding, how much are they able to do on their own, but then I might have them come back to the clinic so that I can then give them a little bit better hands-on education.*

Hybrid practice affords convenience, opportunities for both hands-on treatment, and monitoring the progress of home programs. It serves the needs of sick children and children who live far away from the clinic. It removes the fear of undue burden on the family from the decision to continue therapy to a point of certainty that it is no longer needed.

## Discussion

The change in PT practice over the past years to embrace relationship-driven, family-centered models of care that incorporate a coaching (non-directive) approach to intervention (Plack, 2005) worked to enhance the intellectual acceptance of telePT by therapy professionals in this study. Acceptance was also facilitated by the quality and reliability of the public internet infrastructure across which participants delivered care. In contrast to other reports around the world (Chivate et al., 2022), neither they, nor the families they worked with, regardless of socio-economic factors, experienced problems with basic internet connectivity that would have constituted an essential barrier to distance interaction. Kory’s noted that the technology underlying telePT was easy to learn and operate, highly familiar to her, but *how* to deliver therapy in the small interactive window afforded by a computer, or often, a cell phone was another matter. The circumstance of technological adequacy is a necessary precondition for uncovering the more subtle factors of distance interaction that add to or detract from the quality of care the modality affords.

The most patent deficit of telePT, the fact that therapists could not reach through the screen to get hands-on contact for either child assessment or parental skills verification, was a recurring side-note in therapists’ reflection on their experience with providing care at a distance. While not diminishing the importance of touch in, most specifically, pediatric therapy (Sørvoll et al., 2022), it is testimony to the salience of the practice benefits of tele that therapists were able to set the no-hands-on problem aside and consider what advantages might be inherently mediated by the distance, vs. in-person, mode of interaction. The urgency to adapt the practice to the exigencies of the pandemic paved the way for the acceptance of providing care, at least in the short-term, without reliance on the tactile and kinesthetic. This forced exercise, assuming it could be done at all, could not but have had the effect of strengthening a therapist’s skills in observation and articulation and, consequently, his/her maturation in effecting relationship-driven, family-centered care. Most of our therapist informants, being presented with a technological scenario where they *could* interact with a family at a distance, subsequently learned what was needed to optimize the care they provided.

Enhanced observation and its reciprocal activity, querying, are the first steps in the coaching process described by several participants as fundamental to building the therapist–parent relationship. Therapists used the term “coaching” with a sense that its meaning was understood, but subsequently interspersed words like “teach” and “instruct” in their descriptions of their practice, suggesting that their concepts of coaching were intuitive, vs. grounded in an articulated, reference framework. This usage reflects the literature describing the ambiguity surrounding exactly what coaching is (Ives, 2008) and how it relates to conducting therapy with children and their families (Baldwin et al., 2013; Akhbari Ziegler et al., 2019; Akhbari Ziegler and Hadders-Algra, 2020). Ives (2008) describes conflicting paradigms of coaching as falling across three principal dimensions: directive or non-directive, personal development or goal-focused, and therapeutic or performance-driven. Interestingly, among Ives’ contentions is that coaching is intended for a non-clinical population. Indeed, there is considerable variation in the interpretation of what coaching is and how it is implemented across the various transdisciplinary pediatric rehabilitation frameworks (Akhbari Ziegler et al., 2019). A coaching model for telePT could very well exclude the hands-on component of PT that, according to the informants in this current study, both therapists and patients expect. Defining a model of coaching that includes hands-on interaction can be left for clinical professionals to refine.

TelePT would seem to naturally channel and reinforce the development of observation and questioning skills, suggesting that technologies facilitating telePT may be more conducive to relationship building than traditional in-person clinical interactions. Colleen’s speculation about the intimacy that is suggested by the proximity of participants’ faces to the screen during a telePT session suggests just this characteristic. This perception, however, may arise uniquely out of the typical format for in-clinic therapy where therapists work individually with children, but in the presence of other therapists, patients, and families in a high-activity, gym-like setting. In the context of providing psychological therapies over the Internet, it has been observed that, although visual and auditory stimuli are the ones human beings process consciously, other sensory stimuli also impact affective response (Levey, 2021). By implication, the screening out of these unconscious stimuli may either enhance or detract from the experience and effectiveness of therapy over telehealth applications. Collen’s narrative further identifies the clinic as a public place where individuals are naturally reserved and on their guard in contrast to the home, over cellphone, tablet, or PC, where interactions may be felt as safe and private. Contrasting the typically public and open therapy gym environment with the typically private and confidential psychotherapy environment, it seems evident that the particulars of the in-person to virtual transition determine whether the trade is desirable or not and consequently, whether the therapeutic alliance is enhanced or degraded.

This conjecture of the intimacy of tele-technologies, however, is at odds with Megan’s experience of the difficulty of connecting with a person over media when an initial, in-person connection has not been made. Megan’s observation is consistent with that noted, again, in the recent distance psychotherapy literature which observes that empathic attunement is diminished over tele (Ingram, 2021). This divergence in perception may be a function of technology habituation. Sofia reflected on the difference she observed in her excellent ability to connect with children who were habitually “Face Timing with grandparents” vs. her poor ability to connect with children who had no such exposure. Virtual applications of psychotherapy/analysis for children are nascent research with many questions as to effectiveness (and appropriateness) yet to be explored (Bomba et al., 2021). It would seem that the target of therapy, psychological or physical, will be an important differentiator and should be a part of future research.

Megan also made the observation that the all-important therapist–patient connection was essential to the client’s trust that PT services could, in fact, be adequately delivered. Therapists may see their own reservations about the gaps in services that can be provided *via* telePT reflected in their patients’ reservations about embracing the modality. The disparity in response to proffered partnership, parity, and empowerment illustrated by Blake’s students’ parents and Colleen’s patient’s nanny suggests that technology is not the limiting factor in telePT. The problem for Blake’s marginalized families was not that they did not have access to or know how to use widely disseminated technologies, such as Zoom. They were not comfortable venturing into the therapy domain. Colleen’s patient’s nanny, however, jumped at the chance for involvement.

The ability telePT afforded to “see into the child’s environment” emerged arguably as the greatest value of the modality in patient care. This finding is novel and has implications for both PT process improvement and practitioners’ professional growth. It revealed to therapists so much that they did not know about their patients’ progress and, more strikingly, had not realized they did not know. TelePT provides a unique window into the child’s functioning in the hours he is not in therapy, that is to say, during the vast majority of his time. Our participants’ experiment with telePT hazarded by the COVID-19 shutdown pointed up where therapy conceived in the clinic failed to have an impact on the child’s home environment. It drove home the absolute necessity of fitting the child’s therapy to the circumstances of his daily life and suggests a re-thinking of the current division between interactive therapy services and the design and implementation of the HEP, home exercise program, a cornerstone of therapy for children with chronic conditions.

It was very clear from therapists’ narratives that telePT cannot take place for a young child without the partnership of his/her parent or surrogate (such as the remarkable nanny Sofia described). Young children do not interact on cue; facilitating, partnership with the therapist, is required. Even with bidirectional video for teaching parents therapy skills, the frustration described in the struggles of both therapists and parents in working with interaction restricted to only visual and spoken directions suggests there is an ongoing need for some in-person interaction even under the non-directive paradigm. This observation suggests that a return to patterns of social behavior common prior to the rise of the pandemic, i.e., where parents go to work and children go to school and their days are spent apart, may decrease the demand for pediatric telePT significantly. It will be interesting to see how pediatric PT finds a new steady state, ideally incorporating both the benefits of in-person and hands-on and the check on the ecological validity of prescribed therapy provided by telePT’s view into the home.

Given its potential in parent–therapist relationship building, assuring the ecological validity of therapy programs, and the empowerment of families who seek it, telePT is likely to be part of the future of PT and at least one driver of its evolution, as Kathleen articulated. TelePT has demonstrated too much utility to be set aside as society settles into a new normal post-pandemic. As Colleen describes, there is a compelling case to retain telePT modalities offering them alongside in-person formats for convenience, safety, and service quality enhancement.

## Limitations

This study has several limitations. The sample size is small and the experiences of telePT shared skew to the positive. All participants were highly developed professionally and working in infrastructure environments where neither they nor the families they worked with, regardless of socio-economic factors, experienced any significant problems with basic internet connectivity that would have constituted an essential barrier to distance interaction. The experience of Blake, the therapist working in the public school system, reflects a context for telePT that is quite different from that described by therapists working in a clinical environment. Further exploration of the school-based context for distance therapy is warranted. Finally, the analysis was secondary, meaning we queried static transcripts to further expose the theme of professional growth in the relationship-driven, family-centered practice model we did not expect at the time we conducted therapist interviews. Though a first draft of the results was presented to therapists and they were asked to elaborate, there was no synchronous interaction over possible misunderstandings or missed subtitles.

## Key points for clinical practice

TelePT can be a very useful adjunct to standard, in-clinic PT practice. Most particularly, it provides a check on the ecological validity of how a child’s therapy program is actually being carried out in the child’s day-to-day life. TelePT provides an ideal environment for putting the relationship-driven, family-centered model into practice, scaffolding both professional skills development and promoting parental/family empowerment as increasingly effective partners in realizing therapy objectives for the child.

## Data availability statement

The de-identified raw data supporting the conclusions of this article will be made available by the authors, without undue reservation.

## Ethics statement

The studies described in this article involved human participants and were reviewed and approved by both the IRBEAR of Children’s National Hospital and the Capella University Institutional Review Board. The participants provided their written informed consent to participate in this study. All participants have been pseudonymized and potentially identifiable data excluded. There are no images used in this article.

## Author contributions

EC, MS, and YA: conceptualization, methodology, and review and editing. EC and MS: formal analysis, investigation, resources, data curation, and writing. MS and YA: supervision. All authors have read and agreed to the published version of the article.

## Footnotes

1
https://explore.zoom.us/en/products/meetings/

## References

[B1] Akhbari Ziegler S. Dirks T. Hadders-Algra M. (2019). Coaching in early physical therapy intervention: The COPCA program as an example of translation of theory into practice. *Disabil. Rehabil.* 41 1846–1854. 10.1080/09638288.2018.1448468 29544356

[B2] Akhbari Ziegler S. Hadders-Algra M. (2020). Coaching approaches in early intervention and paediatric rehabilitation. *Dev. Med. Child Neurol.* 62 569–574. 10.1111/dmcn.14493 32065385 PMC7187136

[B3] Baldwin P. King G. Evans J. McDougall S. Tucker M. A. Servais M. (2013). Solution-focused coaching in pediatric rehabilitation: An integrated model for practice. *Phys. Occup. Ther. Pediatr.* 33 467–483. 10.3109/01942638.2013.784718 23611310

[B4] Bomba M. Alibert J.-F. Velt J. (2021). Playing and virtual reality: Teleanalysis with children and adolescents during the COVID-19 pandemic. *Int. J. Psycho Anal.* 102 159–177. 10.1080/00207578.2021.1876401 33952012

[B5] Camden C. Pratte G. Fallon F. Couture M. Berbari J. Tousignant M. (2020). Diversity of practices in telerehabilitation for children with disabilities and effective intervention characteristics: Results from a systematic review. *Disabil. Rehabil.* 42 3424–3436. 10.1080/09638288.2019.1595750 30978110

[B6] Chivate S. Sharma M. Shaikh A. Satarkar C. (2022). Benefits and challenges of telerehabilitation use by pediatric physiotherapists during the COVID-19 pandemic in western and southern India: A cross-sectional survey. *Int. J. Telerehabil.* 14:e6466. 10.5195/ijt.2022.6466 35734383 PMC9186904

[B7] Choong E. A. (2022). Remote versus face-to-face physical therapy services: Health administration and physical therapist perspectives [Doctor of Healthcare Administration Capstone Project].

[B8] Daube Fishman G. Elkins J. (2022). COVID-19 lessons from the field: Toward a pediatric physical therapy telehealth framework. *Int. J. Telerehabil.* 14:e6448. 10.5195/ijt.2022.6448 35734385 PMC9187032

[B9] Dodd K. J. Taylor N. F. Damiano D. L. (2002). A systematic review of the effectiveness of strength-training programs for people with cerebral palsy. *Arch. Phys. Med. Rehabil.* 83 1157–1164. 12161840 10.1053/apmr.2002.34286

[B10] Doraiswamy S. Abraham A. Mamtani R. Cheema S. (2020). Use of telehealth during the COVID-19 pandemic: Scoping review. *J. Med. Internet Res.* 22:e24087. 10.2196/24087 33147166 PMC7710390

[B11] Dostie R. Gaboury I. Cinar E. Camden C. (2022). Acceptability of pediatric telerehabilitation interventions provided by physical therapists and occupational therapists-a scoping review. *Phys. Occup. Ther. Pediatr.* 42 615–634. 10.1080/01942638.2022.2064203 35440285

[B12] Gannotti M. E. (2017). Coupling timing of interventions with dose to optimize plasticity and participation in pediatric neurologic populations. *Pediatr. Phys. Ther.* 29:S37–S47. 10.1097/pep.0000000000000383 28654476 PMC5488702

[B13] Hall J. B. Luechtefeld J. T. Woods M. L. (2021). Adoption of telehealth by pediatric physical therapists during COVID-19: A survey study. *Pediatr. Phys. Ther.* 33 237–244. 10.1097/PEP.0000000000000817 34323864 PMC8478094

[B14] Hsu N. Monasterio E. Rolin O. (2021). Telehealth in pediatric rehabilitation. *Phys. Med. Rehabil. Clin. North Am.* 32 307–317. 10.1016/j.pmr.2020.12.010 33814060

[B15] Ingram D. H. (2021). psychodynamic psychiatry and the therapeutic space in the era of COVID-19. *Psychodyn. Psychiatry* 49 441–452. 10.1521/pdps.2021.49.3.441 34478323

[B16] Ives Y. (2008). What is “coaching”? an exploration of conflicting paradigms. *Int. J. Evid. Based Coach. Mentor.* 6 100–113.

[B17] Levey E. J. (2021). Analyzing from home: The virtual space as a flexible Container. *Psychodyn. Psychiatry* 49 425–440. 10.1521/pdps.2021.49.3.425 34478328

[B18] Libin A. V. Schladen M. M. Danford E. Scholten J. (2016). “Telemedicine in delivering care,” in *Handbook of Psychosocial Interventions for Veterans and Service Members: A Guide for the Non-Military Mental Health Clinician*, eds Ainspan N. D. Bryant C. Penk W. (Washington, D.C: U.S. Department of Veterans Affairs).

[B19] Lillo-Navarro C. Medina-Mirapeix F. Escolar-Reina P. Montilla-Herrador J. Gomez-Arnaldos F. Olivera-Sousa S. L. (2015). Parents of children with physical disabilities perceive that characteristics of home exercise programs and physiotherapists’ teaching styles influence adherence: A qualitative study. *J. Physiother.* 61 81–86. 25804826 10.1016/j.jphys.2015.02.014

[B20] Lord C. Rapley T. Marcroft C. Pearse J. Basu A. (2018). Determinants of parent-delivered therapy interventions in children with cerebral palsy: A qualitative synthesis and checklist. *Child Care Health Dev.* 44 659–669. 10.1111/cch.12592 30033521

[B21] Novak I. (2014). Evidence-based diagnosis, health care, and rehabilitation for children with cerebral palsy. *J. Child Neurol.* 29 1141–1156. 24958005 10.1177/0883073814535503

[B22] Olson C. A. McSwain S. D. Curfman A. L. Chuo J. (2018). the current pediatric telehealth landscape. *Pediatrics* 141:e20172334. 10.1542/peds.2017-2334 29487164

[B23] O’Neil M. E. Fragala-Pinkham M. A. Westcott S. L. Martin K. Chiarello L. A. Valvano J. (2006). Physical therapy clinical management recommendations for children with cerebral palsy—spastic diplegia: Achieving functional mobility outcomes. *Pediatr. Phys. Ther.* 18 49–72. 10.1097/01.pep.0000202099.01653.a9 16508534

[B24] Plack M. (2005). Human nature and research paradigms: Theory meets physical therapy practice. *Qual. Rep.* 10 223–245. 10.46743/2160-3715/2005.1847

[B25] Rabatin A. E. Lynch M. E. Severson M. C. Brandenburg J. E. Driscoll S. W. (2020). Pediatric telerehabilitation medicine: Making your virtual visits efficient, effective and fun. *J. Pediatr. Rehabil. Med.* 13 355–370. 10.3233/PRM-200748 33136081

[B26] Schladen M. M. Koumpouros Y. Choong E. A. Belschner J. L. (2022). “Interactive Computer Play in the Pursuit of Gait Optimization for Children With Cerebral Palsy: Home, Video Games, and Motivation,” in *Assistive technologies for Assessment and Recovery of Neurological Impairments*, ed. Stasolla F. (Hershey, PA: IGI Global).

[B27] Shigekawa E. Fix M. Corbett G. Roby D. H. Coffman J. (2018). the current state of telehealth evidence: A rapid review. *Health Aff.* 37 1975–1982. 10.1377/hlthaff.2018.05132 30633674

[B28] Smith J. A. Flowers P. Larkin M. (2009). *Interpretative Phenomenological Analysis: Theory, Method, and Research.* Thousand Oaks, CA: Sage.

[B29] Sørvoll M. Øberg G. K. Girolami G. L. (2022). the significance of touch in pediatric physiotherapy. *Front. Rehabil. Sci.* 3:893551. 10.3389/fresc.2022.893551 36189075 PMC9397783

[B30] Steenbergen B. van der Kamp J. Verneau M. Jongbloed-Pereboom M. Masters R. S. (2010). Implicit and explicit learning: Applications from basic research to sports for individuals with impaired movement dynamics. *Disabil. Rehabil.* 32 1509–1516. 10.3109/09638288.2010.497035 20575752

[B31] Straus S. E. Tetroe J. Graham I. (2009). Defining knowledge translation. *CMAJ* 181 165–168. 10.1503/cmaj.081229 19620273 PMC2717660

[B32] Taylor N. F. Dodd K. J. McBurney H. Graham H. K. (2004). Factors influencing adherence to a home-based strength-training programme for young people with cerebral palsy. *Physiotherapy* 90 57–53.

